# Agreement, Reliability, and Concurrent Validity of an Outdoor, Wearable-Based Walk Ratio Assessment in Healthy Adults and Chronic Stroke Survivors

**DOI:** 10.3389/fphys.2022.857963

**Published:** 2022-06-20

**Authors:** Simone K. Huber, Ruud H. Knols, Jeremia P. O. Held, Tom Christen, Eling D. de Bruin

**Affiliations:** ^1^ Physiotherapy and Occupational Therapy Research Centre, Directorate of Research and Education, University Hospital Zurich, Zurich, Switzerland; ^2^ Institute of Human Movement Sciences and Sport, Department of Health Sciences and Technology, ETH Zurich, Zurich, Switzerland; ^3^ Department of Physiotherapy and Occupational Therapy, University Hospital Zurich, Zurich, Switzerland; ^4^ Vascular Neurology and Neurorehabilitation, Department of Neurology, University Hospital and University Zurich, Zurich, Switzerland; ^5^ Rehabilitation Center Triemli Zurich, Valens Clinics, Zurich, Switzerland; ^6^ Division of Physiotherapy, Department of Neurobiology, Care Sciences and Society, Karolinska Institute, Stockholm, Sweden; ^7^ Department of Health, OST—Eastern Swiss University of Applied Sciences, St. Gallen, Switzerland

**Keywords:** gait analysis, wearables, walk ratio, cognition, stroke, agreement, reliability, validity

## Abstract

**Purpose:** The walk ratio (WR)—the step-length/cadence relation—is a promising measure for gait control. GPS-running watches deliver clinically relevant outcomes including the WR. The aim of this study was to determine test-retest agreement, reliability and concurrent validity of an outdoor WR assessment using a GPS-running watch.

**Methods:** Healthy adults and moderate—high functioning stroke survivors (≥6 months), performed the 1 km-outdoor walk twice using a GPS-running watch (Garmin Forerunner 35, GFR35) and a Step Activity Monitor (SAM 3). Global cognition was assessed using the Montreal Cognitive Assessment. Test-retest agreement and reliability were assessed using Bland-Altman plots, standard error of measurement (SEM), intraclass correlation coefficients (ICCs) and smallest detectable changes (SDCs). Concurrent validity was determined by the mean difference (MD), standard error (SE), mean absolute percentage errors (MAPEs) and Spearman’s Rho between GFR35 and SAM3. WR values of the two groups were compared by a Welch’s test. A hierarchical multiple regression was performed with the WR as dependent variable and possible predictors as independent variables.

**Results:** Fifty-one healthy adults [median: 60.0 (47.0, 67.0) years) and 20 stroke survivors [mean: 63.1 (12.4) years, median: 76 (30, 146) months post-stroke] were included. Test-retest agreement and reliability were excellent (SEM% ≤ 2.2, ICCs > 0.9, SDC% ≤ 6.1) and concurrent validity was high (MAPE < 5, *ρ* > 0.7) for those walking ≥ 1 m/s. Walking < 1 m/s impaired accurate step counting and reduced agreement, reliability, and validity. The WR differed between healthy adults and stroke survivors (t = −2.126, *p* = 0.045). The hierarchical regression model including stroke and global cognition (Montreal Cognitive Assessment, 0—30) explained 25% of the WR variance (ΔR^2^ = 0.246, *p* < 0.001). Stroke had no effect (*β* = −0.05, *p* = 0.682), but global cognition was a predictor for an altered WR (*β* = 0.44, *p* = 0.001).

**Discussion:** The outdoor WR assessment using the GFR35 showed excellent test-retest agreement, reliability and concurrent validity in healthy adults and chronic stroke survivors walking at least 1 m/s. As the WR seems relevant in chronic stroke, future studies should further investigate this parameter.

## Introduction

Gait analysis is important for the diagnosis and treatment of stroke and in ageing (Lord et al., 2013; Chen et al., 2016). For instance, by informing about fall risk, gait analysis can help prevent falls in older adults (Bergen et al., 2016). In stroke rehabilitation, gait analysis is used to determine and quantify deviations from a normal walking pattern, to support the choice of rehabilitation interventions, and to track the success thereof (Olney and Richards, 1996; Bowden et al., 2012; Wonsetler and Bowden, 2017). Formerly, gait analysis was limited to in-clinic assessments as expensive and cumbersome tools were necessary (Chen et al., 2016). Moreover, these in-lab gait analyses were limited to few gait cycles performed over a short distance. Such standard clinical assessments give information on the capacity level of individuals after a stroke (Tarvonen-Schroder et al., 2015). New tools for gait analysis overcoming some of these limitations are so-called “wearables”: portable, more practicable and affordable sensor-based systems that can provide useful and objective information regarding gait measures also in unsupervised real-life performance (Silva et al., 2015; Chen et al., 2016; Del Din et al., 2016). Wearables allow gait analysis in real-life settings and outdoors (Benson et al., 2018). This is important, as indoor walking assessed under a controlled experimental condition is not necessarily related to real life walking in more natural environments (Tao et al., 2012; Mate and Mayo, 2020). Gait analysis performed in outdoor settings, despite measuring gait under less standardised conditions, can add valuable and ecologically more valid information about a person’s mobility performance (Lord et al., 2004; Silva et al., 2015; Hillel et al., 2019; Mate and Mayo, 2020). To be used with confidence in clinical settings with stroke survivors, however, studies investigating the agreement, reliability, and validity of electronic wearable devices for gait analysis in community settings are needed (Peters et al., 2021).

The walk ratio (WR) is a disability-sensitive index of neuro-motor control of gait that presumably is a promising outcome measure for treatments targeting improvements in motor coordination (Rota et al., 2011). The WR reflects the step-length/cadence relation (Bogen et al., 2018). Evidence suggests that the WR remains constant over different walking speeds and throughout healthy ageing, which makes it a suitable parameter for inter-individual comparisons in populations walking at different preferred walking speeds (Egerton et al., 2011; Bogen et al., 2018). Moreover, there may exist a “normal” WR range indicating healthy walking [evidence suggests an approximate range of 0.58–0.63 cm/step/min, (Bogen et al., 2018)]. In pathological contexts, however, the WR seems to be altered (Kalron, 2016; Norvang et al., 2020). For instance, patients with subacute stroke or multiple sclerosis show lower WR values [0.53—0.58 cm/step/min, (Kalron, 2016; Kalron et al., 2020; Norvang et al., 2020)]. The WR, thus, may be indicative of impaired and/or recovering gait (Suzuki et al., 1999; Kalron, 2016).

Approximately fifty percent of people surviving a stroke recover so far that they retain only minor consequences from the stroke (FragileSuisse, 2022). Such moderate to high functioning stroke survivors may have regained gait ability and speed to be able to ambulate without walking aid and in the community (Faria-Fortini et al., 2019; Peng et al., 2020; Vive et al., 2021), however, still show impaired gait control (Guzik et al., 2017). For measuring gait control in these individuals, the WR may be a helpful parameter, as it adds valuable information regarding gait quality to clinical gait assessments such as gait speed (Bogen et al., 2018). Nevertheless, the WR has so far rarely been measured in chronic stroke survivors (Huber et al., 2021).

The WR can reliably be measured under laboratory conditions in stroke patients (Sekiya and Nagasaki, 1998) and can be captured outside the laboratory *via* satellite positioning (Global Positioning System, GPS) in healthy adults (Terrier and Schutz, 2003). GPS-running watches theoretically deliver clinically relevant outcomes including step length and cadence. These so-called “smart watches” have repeatedly been found to measure step count reliably and validly over slow to fast walking speeds in healthy participants (Evenson and Spade, 2020; Fuller et al., 2020). However, GPS running watches have so far not been assessed to measure the WR while walking outdoors in persons with stroke (Allet et al., 2010; Evenson and Spade, 2020).

The aims of this study were to determine 1) test-retest agreement and reliability as well as, 2) concurrent validity and agreement compared to a gold standard, of outdoor WR assessments performed with a GPS-running watch in healthy adults and chronic stroke survivors. Moreover, to investigate the relevance of the WR in chronic stroke, WR values of healthy adults and chronic stroke survivors, collected with the gold standard, were compared and possible predictor values of an altered WR in healthy adults and chronic stroke survivors were identified.

## Materials and Methods

### Procedures

This was a cross-sectional observational study following a test-retest design in a single study appointment while adhering to GRRAS guidelines for reporting (Kottner et al., 2011). Possible participants were recruited via advertisements and therapists. Subsequently, instructed human movement scientists performed the screening and informed about the study’s aims, benefits and risks *via* phone call. Interested, potential participants received information *via* email and a study appointment. At the appointment, participants first provided written consent, before any other study-specific actions were performed. Demographics and health information were collected, and the cognitive screening was administered (see “Instruments and Measurements”). Subsequently, the outdoor WR assessment was administered twice, following a level-surface outdoor route of 1 km without stairs, which had to be completed in comfortable walking speed. This distance is reflective of community walking in Switzerland, as about 80% of journey stages made on foot are approximately 1 km (Martin-Diener, 2008; Martin-Diener and Martin, 2009). Especially older or mildly impaired pedestrians regularly walk distances of around 1 km, which substantially contributes to independence, health and social integration (Martin-Diener, 2008; Martin-Diener and Martin, 2009). The route followed a 1 km-running track in a public park, where quick direction or speed changes and stops could be avoided as the route included no junctions. No pedestrians or vehicles would cross this track. Participants were instructed to walk with a constant pace and avoid quick turns. The first walking round was measured concurrently with a Garmin Forerunner 35 (GFR35, compare “2.3 Instrument and Measures”) and the Step Activity Monitor (SAM3, compare “2.3 Instrument and Measures”), and the second round with the GFR35 only. The two assessments were separated by a break of at least 20 min. The study was approved by the ethical committee of the Swiss Federal Institute of Technology (ETH) Zurich, Switzerland (Registration No. 2020-N-92).

### Participants and Sample Size Considerations

For this study, healthy adults (≥30 years) and chronic stroke survivors (≥6 months post-stroke, ≥ 30 years) were recruited. Participants were able to walk the 1-km assessment route twice within a two-hours period. Persons who needed a walker for this task (Larsen et al., 2020), who self-reported fall risk or who had experienced a fall within the previous year were excluded. Further exclusion criteria were visible alteration of the gait pattern (e.g., severe claudication, only for healthy participants), a leg prosthesis, cognitive impairment (defined as < 24 Montreal Cognitive Assessment (MoCA) score (Chiti and Pantoni, 2014)), and being at high risk for a serious course of COVID-19 (acute/progressive/terminal disease, chronic respiratory disease, cancer, acute/uncontrolled high blood pressure or diabetes, disease or therapy weakening the immune system, obesity (BMI ≥ 40 kg/m^2^)), as this study was conducted during the pandemic period in 2020–2021. It was planned to recruit at least 50 healthy adults and 20 chronic stroke survivors. For the determination of agreement, reliability and validity, this exceeds the minimum recommended number of participants expecting a moderate or higher correlation (*ρ* ≥ 0.6, compare “Statistical analyses”) and targeting 80% power at a significance level of 0.05 (Bujang and Baharum, 2016; Bujang and Baharum, 2017). Moreover, it meets the recommended number of participants for an exploratory multiple regression analysis with six predictor values [compare ‘2.4 Statistical Analysis, (Van Voorhis and MBL, 2007)].

### Instruments and Measures

This study investigated a GPS-running watch—the Garmin Forerunner 35 (GFR35, Garmin International Ltd., Olathe, KS, United States)—in a 1 km-outdoor WR assessment. The GFR35 holds an accelerometer, a GPS sensor and a heart rate monitor (Garmin, 2021). It was worn on the non-dominant or unaffected wrist. Step count, mean step length and mean cadence were collected from the GFR35 as pre-processed data. To receive values for step length, the GFR35 was set to runner mode. To achieve validation data for step counts, the Step Activity Monitor 3 [SAM3, Cymatech Corporation, Seattle, WA, United States, (Coleman et al., 1999)] was used. The SAM3 has often been used in clinical settings and was found to be accurate, reliable and valid for counting steps in neurological patients and older adults (Resnick et al., 2001; Bowden and Behrman, 2007; Mudge et al., 2007). The SAM3 contains a microprocessor-based accelerometer and allows adjustment of the filtering thresholds for motion and cadence to capture steps in individuals with gait impairment (Macko et al., 2002; Bowden and Behrman, 2007). It was mounted on the ankle on the same body site as the GFR35, using an elastic strap. Online cartographic information (Langstrecken, 2021) and a stopwatch provided distance and time to achieve validation data for mean step length and mean cadence. The online map by “langstrecken.de” is based on Google Maps, which has an imagery resolution of 15 cm (Google, 2014). The measurement track was drawn on the online map multiple times and exact distances from these trials were averaged to get precise validation data for distance. Validation data for mean step length was computed as distance/step count measured by the SAM3. Validation data for mean cadence was computed as total lap time measured by the stopwatch divided by step count measured by the SAM3. For the concurrent measurement in the first walking round, the three tools (GFR35, SAM3 and stopwatch) were carefully synchronized. The SAM3 was programmed to start measuring at a specific time point ahead providing enough time to position the participant for the start of the measurement. As soon as this starting time point arrived, the measurement lap on the GFR35 and the stopwatch were started simultaneously, and the participant started walking. The opposite procedure was applied at the end of the assessment. To collect demographics and health information (including “pain in the lower extremities and back”, compare 2.4 Statistical Analyses), all participants filled out a health questionnaire (Supplementary Figure S1) and to screen for cognitive functions the Montreal Cognitive Assessment (MoCA) was administered. The MoCA has shown high sensitivity and specificity for cognitive impairment including executive functions in chronic stroke and was found to be related to physical performance and functional outcome (Nasreddine et al., 2005; Chiti and Pantoni, 2014). To further describe independence and functional mobility of the stroke sample, the modified Rankin Scale (mRS) and Functional Ambulation Category (FAC) were collected for the participants with stroke (Banks and Marotta, 2007; Mehrholz et al., 2007).

### Statistical Analyses

Mean step length was corrected for body height (mean step length/body height). The WR was calculated as mean corrected step length/mean cadence. Normal distribution of the data was checked using the Kolmogorov-Smirnov test and Shapiro-Wilk test, histograms, and Q-Q-plots and descriptive statistics were determined (mean and standard deviation in case of normally distributed data; median and inter-quartile range otherwise). To determine test-retest agreement, the standard error of measurement (SEM, SEM%) was calculated (de Vet et al., 2006; Kottner et al., 2011) and limits of agreement (LoA) between the two GFR35 measurements were determined using Bland and Altman plots (Bland and Altman, 1986). In case the difference of the two measurements was not normally distributed, the median and 2.5th and 97.5th percentile were used as LoA to draw the plots (Twomey, 2006). To determine relative test-retest reliability, the intraclass-correlation coefficient [ICC(3,k)] with 95% CI was calculated using a two-way mixed-effects model based on mean scores (Weir, 2005; Koo and Li, 2016). An ICC > 0.90 was considered excellent, 0.75–0.90 good, 0.60–0.75 moderate, and < 0.60 low (Portney and Watkins, 2009). To discover absolute test-retest reliability, the smallest detectable change (SDC, SDC%) was calculated (Weir, 2005). To determine concurrent validity, the mean difference (MD) with standard error (SE) and mean absolute percentage error (MAPE) were calculated in case of normally distributed data or the median difference (MdD) with inter-quartile range (IQR) and the median absolute percentage error (MdAPE) in case of non-normally distributed data (Fokkema et al., 2017; Evenson and Spade, 2020). To assess the significance of the MD or MdD, a paired t-test or a Wilcoxon signed-rank test were used. MAPE ≤ 5% was used as cut-off criterion for excellent validity and MAPE ≤ 10% for acceptable validity (Schneider et al., 2004; Tudor-Locke et al., 2006; Evenson and Spade, 2020). Further, correlations between the results of the GFR35 and the SAM3 were examined using Spearman’s Rho, which was interpreted as very strong (*ρ* ≥ 0.8), moderate (0.6 ≤ *ρ* < 0.8), fair (0.3 ≤ *ρ* < 0.6), and poor (*ρ* < 0.3) (Chan, 2003; Akoglu, 2018). To determine agreement with the gold standard, limits of agreement (LoA) between the GFR35 and the SAM3 were determined using Bland and Altman plots (Bland and Altman, 1986). In case the difference of the two measurements was not normally distributed, the same method as described above was applied to account for this.

Due to the unequal sample sizes, the walk ratio values (derived from the SAM3) of the healthy participants and stroke survivors were compared using Welch’s test or a Mann-Whitney-U test, depending on normality of the data (Derrick et al., 2016). To identify predictors of an altered WR, a hierarchical multiple regression with the WR (derived from the SAM3) as dependent variable was performed. The following variables were entered as predictor variables in the model in three blocks: 1) stroke (dichotomous, yes or no) and MoCA score, 2) gait speed < 1 m/s (dichotomous, yes or no) and pain in the lower extremities and back (dichotomous, yes or no), and 3) age and gender. Neurological disorders such as stroke have been reported to alter the WR (Kalron, 2016; Norvang et al., 2020), therefore, we entered the predictor “stroke—yes or no” in the first model. The entry of the MoCA score into the first model was based on growing evidence that cognition plays a role in gait control in older adults and neurological patients (Amboni et al., 2013). Two studies have found correlations between the WR and cognitive functions, especially global cognition, in patients with Multiple Sclerosis and Alzheimer’s Disease, respectively (Kalron, 2016; Knapstad, 2016). Moreover, it is known that cognitive dual-tasking while walking can alter the WR compared to single-task walking (Bogen et al., 2018). The decision to insert ‘gait speed < 1 m/s’ into the second model, despite the WR being reported to be independent of walking speed (Bogen et al., 2018), was based on literature reporting a lower boundary, at which this independency is broken (Murakami and Otaka, 2017). Leg and back pain have been found to alter walking patterns in older adults and chronic lower back patients, respectively (Alexander, 1996; Al-Obaidi et al., 2003; Callisaya et al., 2012). Regarding the WR, however, little evidence indicates that there may be no influence of pain (Thingstad et al., 2015; Carvalho et al., 2019). Therefore, “pain in the lower extremities and back” was entered in the second model. Furthermore, it has been found that the WR is independent of age and gender, which is why these two predictors were entered into the model in the last step. The resulting R^2^-values were tested on significance and the influence of the individual parameters on the model was investigated using correlations and significance. As both, scalar and dichotomous variables were entered into the regression model as predictors, standardized correlation coefficients were reported. The assumptions for a multiple regression were tested using ZRESID/ZPRED plots to check for linearity and homoscedasticity, histograms and Q-Q-plots of the residuals to test normality thereof and calculating the variance inflation factor (VIF) to exclude collinearity of the independent variables (Field, 2013). If any assumptions were not met, robust regression was performed using bootstrapping to achieve bias corrected and accelerated confidence intervals (Field, 2013). Significance was set at *p* < 0.05 and effect sizes were calculated as r (Bravais-Pearson correlation coefficient) and interpreted as small (r < 0.3), medium (0.3 ≤ r < 0.5) and large (0.5 ≤ r) (Field, 2013). All statistical analyses were conducted using SPSS Statistics (version 26 for windows; IBM, Chicago, IL, United States).

## Results

### Participants

**FIGURE 1:**
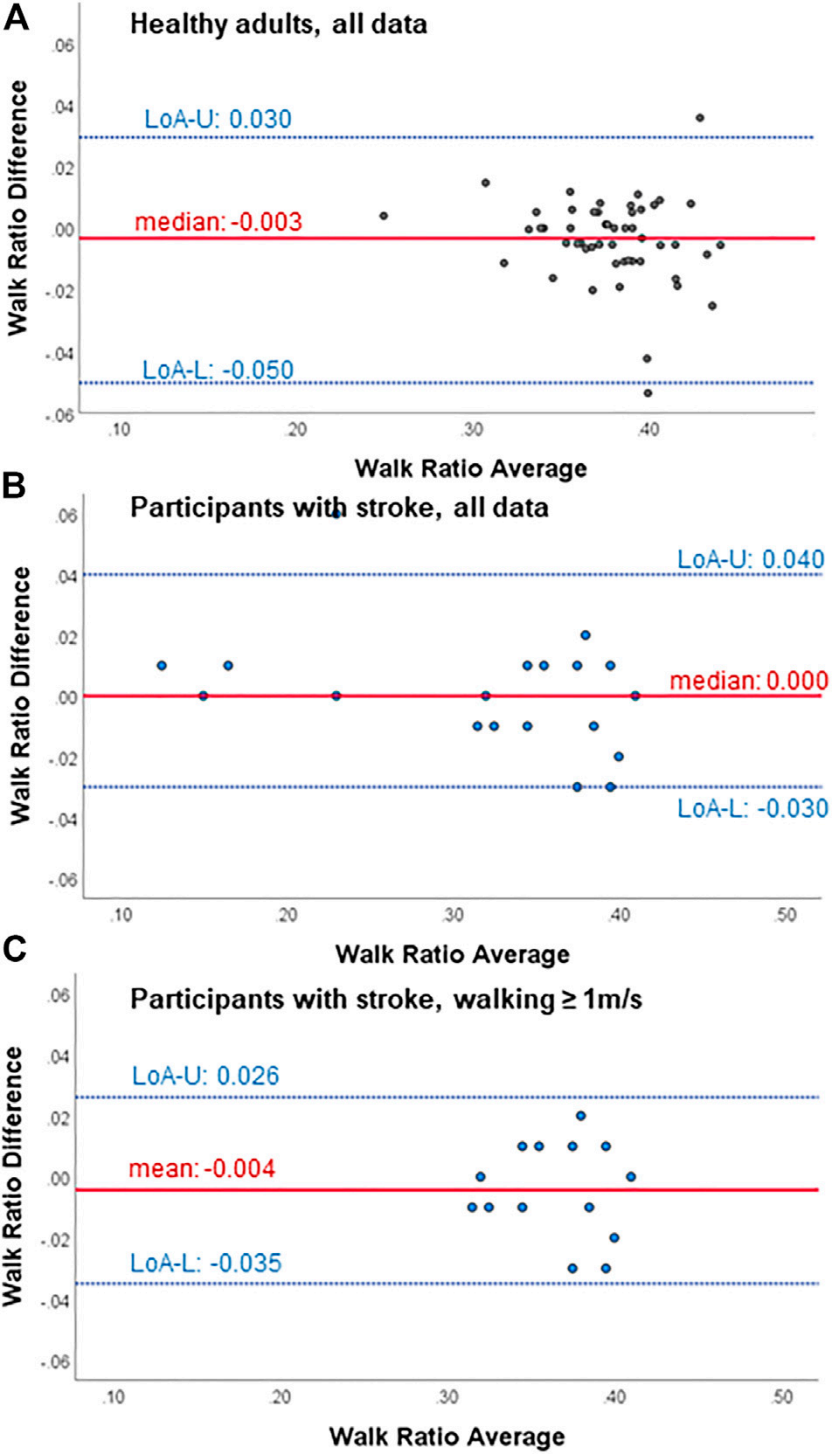
Bland-Altman plots for test-retest agreement of the walk ratio measures from the wearable-based outdoor walk ratio assessment in **(A)** healthy participants (*n* = 51), **(B)** all participant with chronic stroke (*n* = 19) and **(C)** participants with chronic stroke walking ≥ 1 m/s (*n* = 14). Diff, difference; LoA-U, upper limit of agreement; LoA-L, lower limit of agreement.

**FIGURE 2:**
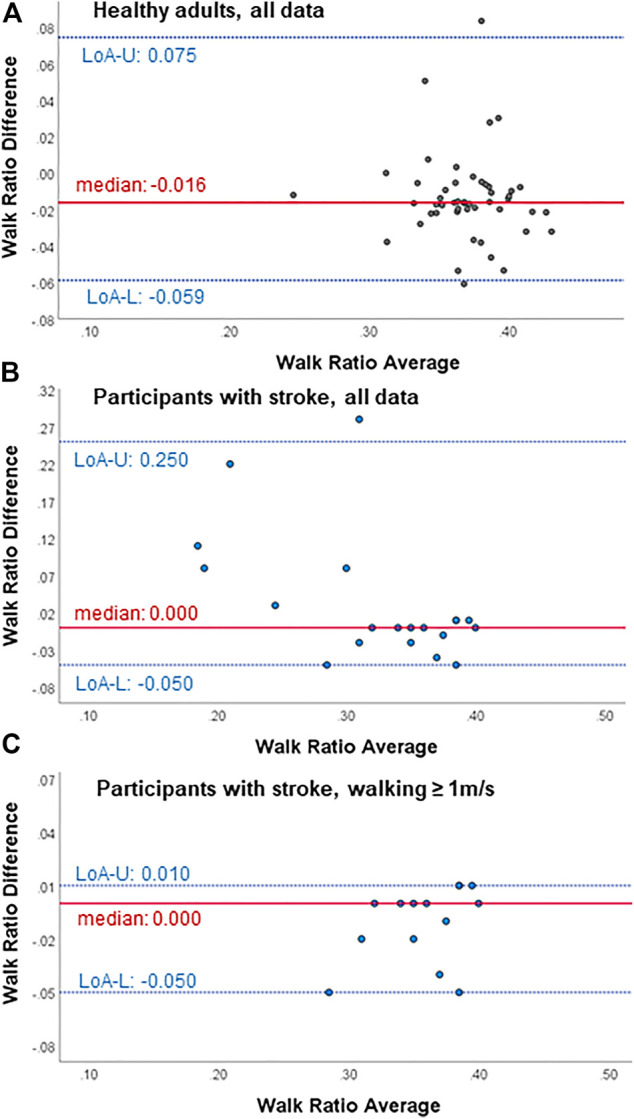
Bland-Altman plots for agreement with the gold standard (SAM3) of the walk ratio from the wearable-based outdoor walk ratio assessment in **(A)** healthy participants (*n* = 50), **(B)** all participants with chronic stroke (*n* = 20) and **(C)** participants with chronic stroke walking ≥ 1 m/s (*n* = 14). Diff, difference; LoA-U, upper limit of agreement; LoA-L, lower limit of agreement.

Fifty-one healthy adults and twenty stroke survivors were included (demographics and characteristics in Table 1). As the SAM3 did not record step count in one healthy adult, 51 healthy adults were analysed for the test-retest analyses and 50 for concurrent validity. Moreover, one participant with stroke decided to leave the study after the first walking round. Therefore, 19 stroke survivors were included for the test-retest analyses and 20 for concurrent validity. For six participants with stroke, the GFR35 failed to record a realistic number of steps (stroke survivors who walked < 1 m/s, Tables 2, 3). Therefore, to evaluate if inaccurate step counting due to slow walking speed (<1 m/s) was the main determinant of reduced agreement, reliability and validity in the stroke group, additional analyses excluding those slow walking participants with stroke were conducted (labelled as C in Tables 2,3; Figures 1, 2). No adverse events occurred during any of the outdoor walking assessments.

**TABLE 1:** Descriptive characteristics.

Descriptives	Unit	Healthy adults (*n* = 51)		Stroke survivors (*n* = 20)		Differences		
		M (SD)/Md [IQR]	Range/N	M (SD)/Md [IQR]	Range/N	t/T	*p*-value	ES
Age	[years]	60.0 [47.0, 67.0]	30.0–81.0	63.1 (12.43)	34.0–85.0	626.00^b^	0.14	0.18
Sex [f/m]			31/20		7/13	-	-	-
Stroke type [ischemic/haemorrhagic/?]		-	-		13/6/1	-	-	-
Affected brain side [left/right]		-	-		9/11	-	-	-
Affected body side [right/left]		-	-		11/9	-	-	-
Time since stroke	[months]	-	-	76 [30, 146]	12–171	-	-	-
Years of Education	[years]	15.95 (2.84)	11–23	13.9 (3.89)	6–21	2.46^a^	0.02*	0.28
MoCA	[n]	29.0 [28.0, 30.0]	26–30	27.0 [24.25, 28.0]	24–30	222.00^b^	<0.01*	0.45
mRS	[n]	-	-	2.0 [1.0, 2.0]	0–3	-	-	-
FAC	[n]	-	-	4.5 [4.0, 5.0]	3–5	-	-	-
Gait Speed	[m/s]	1.28 (0.10)	1.00–1.50	1.34 [0.77, 1.47]	0.38–1.77	593.50^b^	0.29	0.13
Step Count/1 km	[n]	1,451 [1,384, 1,498]	1,308–2033	1,449 [1,283, 1879]	1,161–2,796	497.00^b^	0.97	0.01
Mean Step Length	[cm]	68.92 [66.75, 72.24]	49.18–76.46	65.79 (16.23)	35.77–86.15	503.00^b^	0.97	0.01
Mean Cadence	[steps/min]	112 (6.5)	99–126	120 [104, 124]	67–145	628.50^b^	0.10	0.20
Walk Ratio	[cm/steps/min]	0.616 (0.072)	0.390–0.750	0.587 (0.111)	0.380–0.790	1.15^a^	0.26	0.22

*significant result at p < 0.05.

a t/T, test statistic of Welch’s test.

b Mann-Whitney-U test to compare means of the two groups.

Descriptives for healthy participants (*n* = 51) and participants with chronic stroke (*n* = 20).

M, mean; SD, standard deviation; Md, median; IQR, interquartile range; ES, effect size; r, (Bravais-Pearson correlation coefficient); ?, unknown, information not available; MoCA, Montreal Cognitive Assessment; mRS, modified Rankin Scale; FAC, Functional Ambulatory Category.

**TABLE 2:** Test-retest agreement & reliability.

Parameter	Unit	Agreement					Relative reliability			Absolute reliability	
		MD^a^/MdD^b^	LoA-L	LoA-U	SEM	SEM %	ICC [95% CI]	F	*p*	SDC	SDC %
A		Healthy participants, all data included									
Step Count	[]	−0.7^a^	−36.4	35.0	14.0	1.0	0.988^b^ [0.979, 0.993]	81.52	<0.001^∗^	38.8	3.0
Step Length	[cm]	−0.56^b^	−3.38	2.48	0.67	2.0	0.951^b^ [0.915, 0.972]	20.19	<0.001^∗^	1.84	4.0
Cadence	[steps/min]	0.0^b^	−3.4	5.4	0.9	1.0	0.976^a^ [0.958, 0.986]	40.66	<0.001^∗^	2.6	2.0
Walk Ratio	[cm/steps/min]	−0.003^b^	−0.050	0.030	0.007	2.0	0.958^a^ [0.925, 0.976]	25.15	<0.001^∗^	0.020	5.0
B		Participants with stroke, all data included									
Step Count	[]	8.0^b^	−492.2	246.7	32.4	2.6	0.996^b^ [0.989, 0.998]	216.05	<0.001^∗^	89.9	7.3
Step Length	[cm]	0.11^a^	−3.57	3.79	1.69	4.6	0.975^b^ [0.935, 0.990]	38.24	<0.001^∗^	4.68	12.6
Cadence	[steps/min]	−0.4^a^	−4.4	3.6	0.7	0.6	0.985^a^ [0.961, 0.994]	63.29	<0.001^∗^	2.0	1.7
Walk Ratio	[cm/steps/min]	0.000^b^	−0.030	0.040	0.016	4.9	0.971^b^ [0.924, 0.989]	32.51	<0.001^∗^	0.043	13.7
C		Participants with stroke, walking ≥ 1 m/s									
Step Count	[]	10.3^a^	−41.3	61.9	13.7	1.0	0.989^a^ [0.967, 0.997]	101.05	<0.001^∗^	38.1	2.8
Step Length	[cm]	−0.27^a^	−3.42	2.88	0.81	1.9	0.948^a^ [0.841, 0.983]	18.45	<0.001^∗^	2.24	5.2
Cadence	[steps/min]	−0.1^a^	−3.9	3.7	1.0	0.8	0.969^a^ [0.904, 0.990]	30.42	<0.001^∗^	2.6	2.2
Walk Ratio	[cm/steps/min]	−0.004^a^	−0.035	0.026	0.008	2.2	0.939^a^ [0.816, 0.980]	16.41	<0.001^∗^	0.022	6.1

^∗^significant results p ≤ 0.05.

a parametric statistics.

b non-parametric statistics.

Test-retest agreement and reliability results for healthy participants (*n* = 51) and participants with chronic stroke (all data, *n* = 19 and walking ≥ 1 m/s, *n* = 14). in participants who walked < 1 m/s, the GFR35 recorded no or inaccurate step count.

MD, mean difference; MdD, median difference; LoA-L, lower limit of agreement; LoA-U, upper limit of agreement; SEM, standard error of measurement; ICC, intra-class correlation coefficient; CI, confidence interval; F, test statistic for ICC; SDC, smallest detectable change.

**TABLE 3:** Validity.

Parameter	Unit	Concurrent validity							Agreement		
		MD^a^ (SE)/MdD^b^ [IQR]	t^a^/T^b^	*p*	ES	MAPE^a^/MdAPE^b^	Correlation	*p*	MD^a^/MdD^b^	LoA-L	LoA-U
A		Healthy participants, all data included									
Step Count	[]	8.8 [0.0, 19.6]	104.50^b^	<0.001^∗^	0.46	0.94^b^	0.918	<0.001^∗^	8.8^b^	−133.8	116.9
Step Length	[cm]	1.33 (0.21)	6.64^a^	<0.001^∗^	0.67	3.63^b^	0.869	<0.001^∗^	-1.45^b^	−3.84	3.76
Cadence	[steps/min]	0.6 (0.5)	1.36^a^	0.18	0.19	0.95^b^	0.875	<0.001^∗^	1.1^b^	−11.3	7.3
Walk Ratio	[cm/steps/min]	0.014 (0.003)	4.06^a^	<0.001^∗^	0.50	4.57^b^	0.750	<0.001^∗^	-0.016^b^	−0.059	0.075
B		Participants with stroke, all data included									
Step Count	[]	48.5 [8.8, 880.3]	51.00^b^	0.077	0.28	3.92^b^	0.239	0.14	5.0^b^	−63.7	2,689.5
Step Length	[cm]	1.87 [0.57, 3.65]	48.00^b^	0.033^∗^	0.34	4.20^b^	0.798	<0.001^∗^	1.29^b^	−3.39	11.83
Cadence	[steps/min]	7.2 [2.5, 15.6]	103.00^b^	0.940	0.01	5.76^b^	0.328	0.05^∗^	2.2^b^	−48.8	13.9
Walk Ratio	[cm/steps/min]	0.020 [0.003, 0.073]	40.50^b^	0.267	0.18	6.27^b^	0.581	0.001^∗^	0.000^b^	−0.050	0.250
C		Participants with stroke, walking ≥ 1 m/s									
Step Count	[]	30.1 (8.1)	−0.14^a^	0.891	0.04	2.24^a^	0.824	<0.001^∗^	−1.6^a^	−86.9	83.5
Step Length	[cm]	1.40 (0.29)	0.21^a^	0.839	0.06	3.24^a^	0.648	0.001^∗^	0.10^a^	−3.45	3.66
Cadence	[steps/min]	5.3 (1.3)	3.85^a^	0.002^∗^	0.73	4.15^a^	0.709	0.001^∗^	3.7^b^	−1.2	15.6
Walk Ratio	[cm/steps/min]	0.016 (0.005)	−2.00^a^	0.067	0.48	4.92^a^	0.713	0.001^∗^	0.000^b^	−0.050	0.010

^∗^significant results *p* ≤ 0.05.

a parametric statistics.

b non-parametric statistics.

Concurrent validity and agreement with the gold standard results for healthy participants (*n* = 50) and for participants with chronic stroke (all data, *n* = 20 and walking ≥ 1 m/s, *n* = 14). in participants who walked < 1 m/s, the Garmin watch recorded no or inaccurate step count.

MD, mean difference; SE, standard error; MdD, median difference; IQR, interquartile range; t/T, test statistic of comparing means; ES, effect size: r (Bravais-Pearson correlation coefficient); Correlation, Spearman’s Rho; LoA-L, lower limits of agreement; LoA-U, upper limits of agreement.

### Test-Retest Agreement/Reliability, Concurrent Validity

Within all participants, limits of agreement and Bland-Altman plots speak for high test-retest agreement of the GFR35 (Table 2; Figure 1; Supplementary Figures S2, S4, S6). Test-retest agreement determined by the SEM, however, was only high in healthy adults and stroke survivors walking ≥ 1 m/s (SEM% = 0.8—2.2, Table 2 A,C), while in the analysis including all stroke survivors, the higher SEMs for step count, step length and the WR indicated reduced agreement (SEM % = 2.6—4.9, Table 2 B). The GFR35 showed excellent relative test-retest reliability for all parameters in all participants (ICCs > 0.9, *p* < 0.001, Table 2). In healthy adults and stroke survivors walking ≥ 1 m/s, the absolute test-retest reliability was high (SDC% = 2.0—6.1, Table 2 A,C), while in the analysis including all stroke survivors, higher SDCs for step count, step length and the WR indicated reduced absolute reliability (SDC% = 7.3—13.7, Table 2B). Moreover, excellent concurrent validity was found in healthy adults and chronic stroke survivors walking ≥ 1 m/s represented by low MAPEs (M(d)APE ≤ 5%, Table 3 A,C) and moderate to strong and significant correlations with the SAM3 (0.648 ≤ *ρ* ≤ 0.918, Table 3 A,C). In the analysis including all stroke survivors, the concurrent validity was still acceptable for step length, cadence and the WR (MdAPE ≤ 6.27, *ρ* ≤ 0.328, *p* ≤ 0.05, Table 3 B), however, not for step count (MdAPE = 3.92, *ρ* = 0.239, *p* = 0.14). The comparison of the GFR35 and the SAM3 resulted in both, non-significant and significant effects (Table 3). Limits of agreement and Bland-Altman plots in all parameters speak for high agreement between the GFR35 and the SAM3 in all participants (Table 3; Figure 2, Supplementary S3, S5, S7).

### Walk Ratio in Healthy Adults and Chronic Stroke Survivors

Within the healthy adults, an outlier regarding the WR was detected. It appeared that the outlier was the oldest participant within the healthy group (81 years), whose WR may have been influenced by other unknown pathologies as the WR did also differ from the normal values known from literature (Bogen et al., 2018). Therefore, the value of this outlier was excluded for the comparison of WR values between healthy adults and stroke survivors. In this comparison, stroke survivors showed a lower WR compared to healthy adults (t = −2.126, df = 22.700, *p* = 0.045). All assumptions for the hierarchical multiple regression were met, the results are presented in Table 4. The model with all six predictors resulted in a significant R^2^ value (R^2^ = 0.301, *p* < 0.001), wherein the first model (stroke and global cognition) explained the largest part of WR variance with a highly significant R^2^ change (ΔR^2^ = 0.254, ΔF = 11.560, *p* < 0.001). The factors pain and “gait speed < 1 m/s” explained few further variance (ΔR^2^ = 0.039, ΔF = 1.822, *p* = 0.170), while age and gender did not further explain the WR’s variance (ΔR^2^ = 0.008, ΔF = 0.363, *p* = 0.697). Regarding the predictors, the MoCA score over both populations showed the highest and only significant association to the WR (*β* = 0.444, *p* = 0.001). The second highest associated predictor for the WR with a trend towards significance was “gait speed < 1 m/s” (*β* = −0.221, *p* = 0.088). All other predictors were not significantly associated with the WR.

**TABLE 4:** Predictors of an altered Walk Ratio.

Hierarchical multiple regression analysis											
Model	Predictors	R2	F	df1 of F	df2 of F	*p* of F	ΔR2	ΔF	df1 of ΔF	df2 of ΔF	*p* of ΔF
1	Stroke, MoCA	0.254	11.560	2	68	<0.001∗	0.254	11.560	2	68	<0.001∗
2	Stroke, MoCA, ‘LE-B Pain’, ‘GS < 1 m/s’	0.293	6.830	4	66	<0.001∗	0.039	1.822	2	66	0.170
3	Stroke, MoCA, ‘LE-B Pain’, ‘GS < 1 m/s’, Age, Gender	0.301	4.587	6	64	0.001∗	0.008	0.363	2	64	0.697
**Coefficients**											
**Model**	**Predictors**	**β**	**t**	***p***							
1	Stroke	0.064	0.467	0.642							
	MoCA	0.444	3.324	0.001∗							
2	‘LE-B Pain’	−0.099	−0.904	0.369							
	‘GS < 1 m/s’	−0.221	−1.742	0.074							
3	Age	0.016	0.138	0.891							
	Gender	0.088	0.781	0.437							

*significant result at *p* ≤ 0.05.

Results of the hierarchical multiple regression analysis. R^2^, proportion of explained variance by this model; F, test statistic for significance test of R^2^; ΔR^2^, additional proportion of explained variance by this model; ΔF, test statistic for significance test of ΔR^2^; df1, degrees of freedom Regression; df2, degrees of freedom Residuals; *p*, significance of test statistic; MoCA: Montreal Cognitive Assessment (scores 0–30); “GS < 1 m/s”, gait speed < 1 m/s; “LE-B Pain”, pain in the lower extremities and back; *β*; standardized correlation coefficient between dependent and independent variables in regression model 3; t, test statistic for significance test of *β*.

## Discussion

The aim of this cross-sectional observational study was to determine test-retest agreement and reliability as well as concurrent validity and agreement with a gold standard of an outdoor walk ratio assessment using the Garmin Forerunner 35 (GFR35) in healthy adults and chronic stroke survivors. WR values in healthy adults and stroke survivors were explored and possible predictor values of an altered WR in these groups identified. We found excellent test-retest agreement and reliability as well as excellent concurrent validity and agreement with the gold standard for the outdoor WR assessment in healthy adults and chronic stroke survivors who walked at least 1 m/s. Below this cut-off, the GFR35 revealed problems recording step counts, which negatively affected the agreement, reliability and validity of all parameters. A systematic error between the measurements of the GFR35 and the gold standard implied that the two methods cannot be used interchangeably. Confirming previous results, we found lower WR values in stroke survivors compared to healthy adults. Moreover, global cognition (MoCA) was associated with an altered WR.

### Test-Retest Agreement/Reliability, Concurrent Validity

We found acceptable to excellent test-retest agreement and reliability as well as concurrent validity of the outdoor WR assessment using a GFR35 in healthy adults and stroke survivors who walked at least 1 m/s (Tables 2, 3). Bland-Altman plots showed that no more than 5% of the points lay outside of the limits of agreement in any of the plots (Figures 1, 2, Supplementary Figure S2–7), indicating high agreement between the test and retest as well as between the GF35 and the gold standard in all participants (Bland and Altman, 1999). The results for SEMs and SDCs in the outdoor WR assessment were lower compared to the results of three studies investigating a camera-based gait analyses on the ground in healthy adults and stroke survivors (Latorre et al., 2019; Chaparro-Rico and Cafolla, 2020) and an infrared-based gait analysis system in healthy adults (Hsu et al., 2016). We found SEM% values (<5.0%) as well as SDC% (<14.0%) in all groups and for all parameters, which can be interpreted as acceptable to excellent results regarding test-retest agreement and absolute test-retest reliability (Hsu et al., 2016; Latorre et al., 2019; Chaparro-Rico and Cafolla, 2020). This is in line with previous results in survivors of stroke, where Garmin watches and other wrist-worn wearables showed high reliability and validity for step counting (Schaffer et al., 2017; Evenson and Spade, 2020; Fuller et al., 2020).

We observed that the GFR35 underestimated or even zero-counted steps at slow speeds (<1 m/s), which reduced test-retest agreement (Table 2B), absolute test-retest reliability (Table 2B) and concurrent validity (Table 3B) in participants walking below this cut-off. A plausible reason may be that at slow walking speeds, the natural arm swing during walking tends to be reduced, which may have hindered the GFR35 to detect acceleration at the wrist (Fokkema et al., 2017). Several studies in healthy adults and stroke survivors have reported similar cut-off speeds, below which wrist-worn wearables (including smart watches by Fitbit, Nike and Garmin) failed to accurately count steps (Fulk et al., 2014; Schaffer et al., 2017; Svarre et al., 2020). These cut-offs (between 0.5 and 0.7 m/s) were lower compared to the cut-off found in this study (1.0 m/s), which may be because those other measurements took place under lab-conditions (indoors, in a hallway or on a treadmill) as opposed to the outdoor walking used in our study. Participants may have felt more secure and less distracted under laboratory conditions in those studies as opposed to the outdoor environment in our study, therefore, showing gait patterns closer to normal also at slower walking speeds. This may have included a more pronounced arm swing, resulting in better results at walking speeds between 0.7 and 1.0 m/s.

Parameters apart from step count have rarely been investigated in wrist-worn wearables to date. Only one other study investigated the validity of wrist-worn wearables measuring cadence (Han et al., 2020), however, Han et al. reported real-time cadence, whereas we reported mean cadence of the whole assessment. Nevertheless, their results correspond well with our findings. We noticed that in case steps had not been counted accurately, the GFR35 reported a “default”-cadence of 122 steps/min. The same behaviour of a Garmin Forerunner was reported by Han et al. below an almost identical cut-off speed (<1.1 m/s) (Han et al., 2020). Therefore, our results confirm that the GFR35 may not be feasible for gait analysis in participants walking below 1 m/s. Moreover, the mean difference between the GFR35 and the gold standard was significant for several parameters, indicating a systematic error between the two measurements. This systematic error leads to the conclusion that the two methods cannot be used interchangeably.

### Walk Ratio in Healthy Adults and Chronic Stroke Survivors

Previous findings informed our hypothesis that chronic stroke survivors may have a lower WR than healthy adults (Kalron, 2016; Norvang et al., 2020). Indeed, we found significantly lower WR values in stroke survivors compared to healthy adults, despite our stroke sample being rather well-rehabilitated (Table 1, MoCA, mRS, FAC). The sample was rather well-rehabilitated due to the inclusion criteria, which were driven by 1) the nature of the outdoor WR assessment (the outdoor 1 km-walk) and by 2) the fact that we had to exclude participants at risk for falls to be able to keep a distance during the measurements due to the pandemic situation. The results of the multiple regression, however, showed that only global cognition affected the variance in the WR (*β* = 0.444, *p* = 0.001), while stroke had no effect (*β* = 0.016, *p* = 0.642). This association between the WR and global cognition is in line with other reports of significant correlations found in patients with Multiple Sclerosis and Alzheimer’s Disease (Kalron, 2016; Knapstad, 2016). This corresponds with the theory of Motoric Cognitive Risk syndrome, which has recently been linked to cardiovascular diseases such as stroke (Beauchet et al., 2018). This theory claims that gait and cognitive impairments are intertwined in ageing and neurological populations, and would be in agreement with the fact that walking and cognitive deficits after stroke share structural and functional roots (Verstraeten et al., 2016; Ursin et al., 2019). Moreover, the apparent relationship between cognitive abilities and the WR has been found in previous studies (Rota et al., 2011; Callisaya et al., 2012; Kalron et al., 2020). Motoric Cognitive Risk syndrome includes slow walking speed, which may correspond with our finding that slow gait speed (<1 m/s) showed a tendency of being associated with an altered WR (*β* = -0.219, *p* = 0.07). Generally, the WR is reported to be independent from gait speed (Bogen et al., 2018). However, it has also been reported that a lower limit of this independency may exist (Suzuki et al., 1999; Dean et al., 2001; Nakakubo et al., 2018). Murakami and his team found this lower limit to be at a gait speed of 1.04 m/s, which is close to our finding (Murakami and Otaka, 2017). Finally, we found that the WR was independent of pain in the lower extremities and back, age and gender, which is in accordance with other reports (Thingstad et al., 2015; Bogen et al., 2018; Carvalho et al., 2019). The results of these additional analyses speak for the relevance of WR assessments in chronic stroke survivors. As the GFR35 was found to fail in slow walking individuals, tools such as the SAM3 or other sensors capable of measuring gait parameters outdoors and over longer distances may be used in future studies to measure the WR. Moreover, with the present explorative analysis around the WR, we opened new questions for further research. Future studies may further investigate the relationship between the WR and global cognition as well as the relevance of the WR as a measure of gait control in moderate to high functioning chronic stroke survivors.

### Strengths and Limitations

This study investigated the test-retest agreement and reliability as well as concurrent validity of measuring step count, step length, and cadence with a wrist-worn wearable in healthy adults and chronic stroke survivors. From these parameters, we calculated the height-corrected walk ratio, a relevant yet under-investigated walking parameter in ageing and stroke rehabilitation (Bogen et al., 2018; Norvang et al., 2020). An altered WR may resemble cautious gait and impaired gait control, therefore, WR assessments could help understand the quality of walking in the context of ageing and neurological disorders (Suzuki et al., 1999; Egerton et al., 2011; Rota et al., 2011). The procedures were performed outdoors and over a longer distance compared to many previous studies with such smart watches. Wearables provide the opportunity to transpose walking assessments into outdoor settings, which adds ecologically valid information to understanding gait ability of neurological patients such as with stroke (Peters et al., 2021). We recruited an adequate number of participants for the statistical analyses performed in this study. However, as the GFR35 failed to accurately count steps in six individuals with stroke, the sample size for the agreement/reliability/validity analyses in stroke survivors walking > 1 m/s was smaller than intended. Unequal sample sizes, however, can reduce the power of the statistical tests. We accounted for this by using Welch’s test (Derrick et al., 2016). A further possible limitation of this study is that in both groups, people with reduced mobility, at risk for falls and in need of a walker had to be excluded (compare “Participants and Sample Size Considerations”), therefore, the obtained results cannot be generalised to wider populations of older adults and stroke survivors. Furthermore, the stroke aetiology information is limited as the study team did not have contact to the stroke survivor’s clinicians and, therefore, the participants with stroke self-reported the information available to them.

## Conclusion

The present outdoor walk ratio assessment using a wrist-worn Garmin watch on a 1 km-outdoor route showed good test-retest agreement, was reliable and valid in participants who walked at least 1 m/s. In healthy adults and stroke survivors walking below this cut-off, the Garmin Forerunner 35 may not be feasible for measuring step count, step length and cadence, as it may zero-count or underestimate step count, which falsifies step length and cadence measures. Nevertheless, WR assessments using other tools may be important in chronic stroke survivors. This, as chronic stroke survivors had significantly lower walk ratio values compared to healthy adults. However, in this study population of self-reported healthy adults and moderate to high functioning stroke survivors, stroke was not found to be associated with an altered walk ratio while global cognition was. This opens new research questions for future studies, which may clarify the relation between stroke, the WR and global cognition.

## Data Availability

The original contributions presented in the study are included in the article/Supplementary Material, further inquiries can be directed to the corresponding author.
