# Supplementary material for: Agreement, Reliability, and Concurrent Validity of an Outdoor, Wearable-Based Walk Ratio Assessment in Healthy Adults and Chronic Stroke Survivors
Source: Front Physiol. 2022 Jun 20;13:857963. doi: 10.3389/fphys.2022.857963 (PMC9252290; doi:10.3389/fphys.2022.857963)
Supplement: Supplementary file 1 [file DataSheet2.PDF]

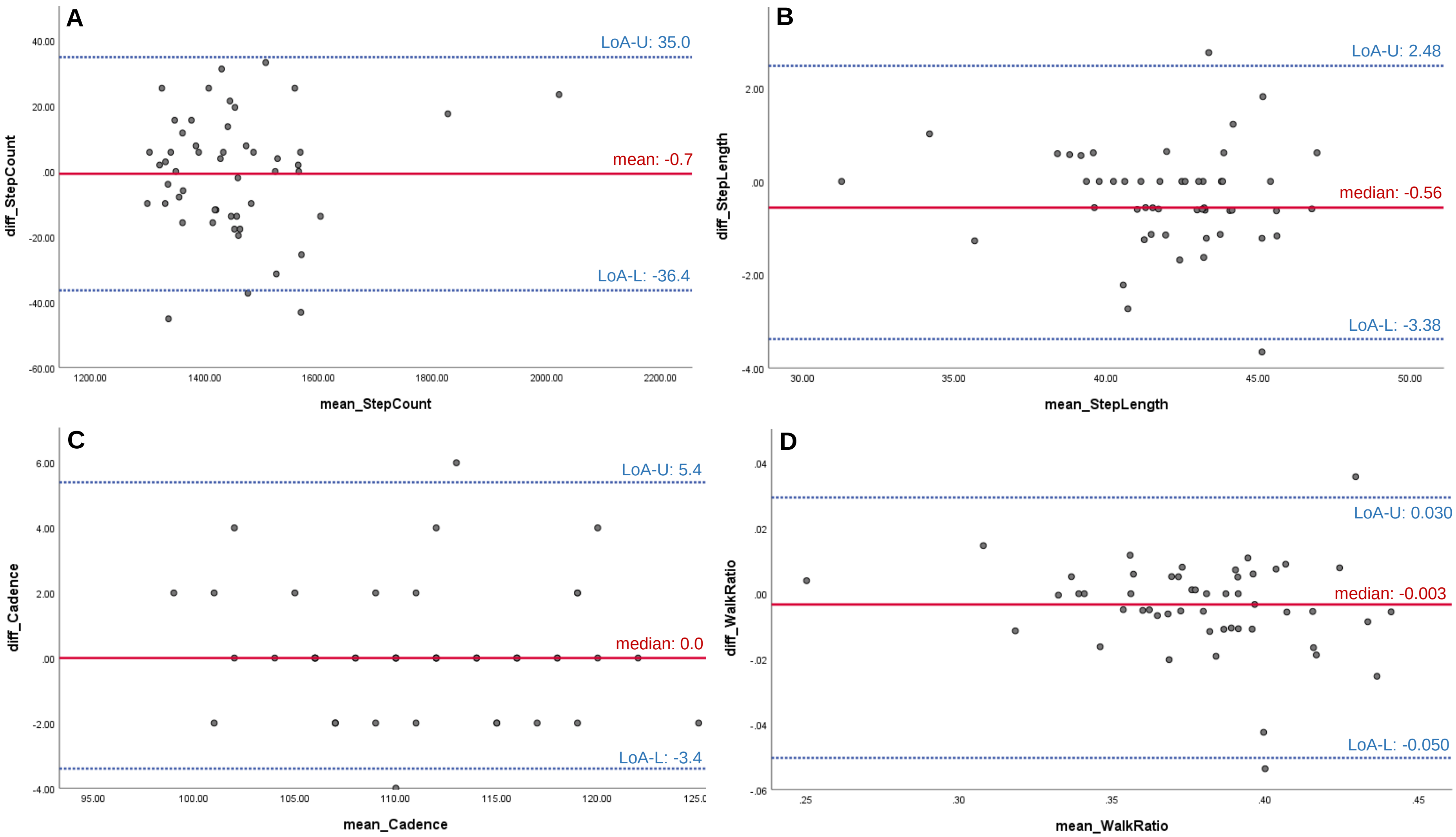

Supplement 2:
Bland-Altman Plots for Test-retest Agreement in Healthy Participants (N = 51); A: Step Count, B: Step Length, C: Cadence, D: Walk Ratio
diff: difference; LoA-U: upper Limit of Agreement; LoA-L: lower Limit of Agreement

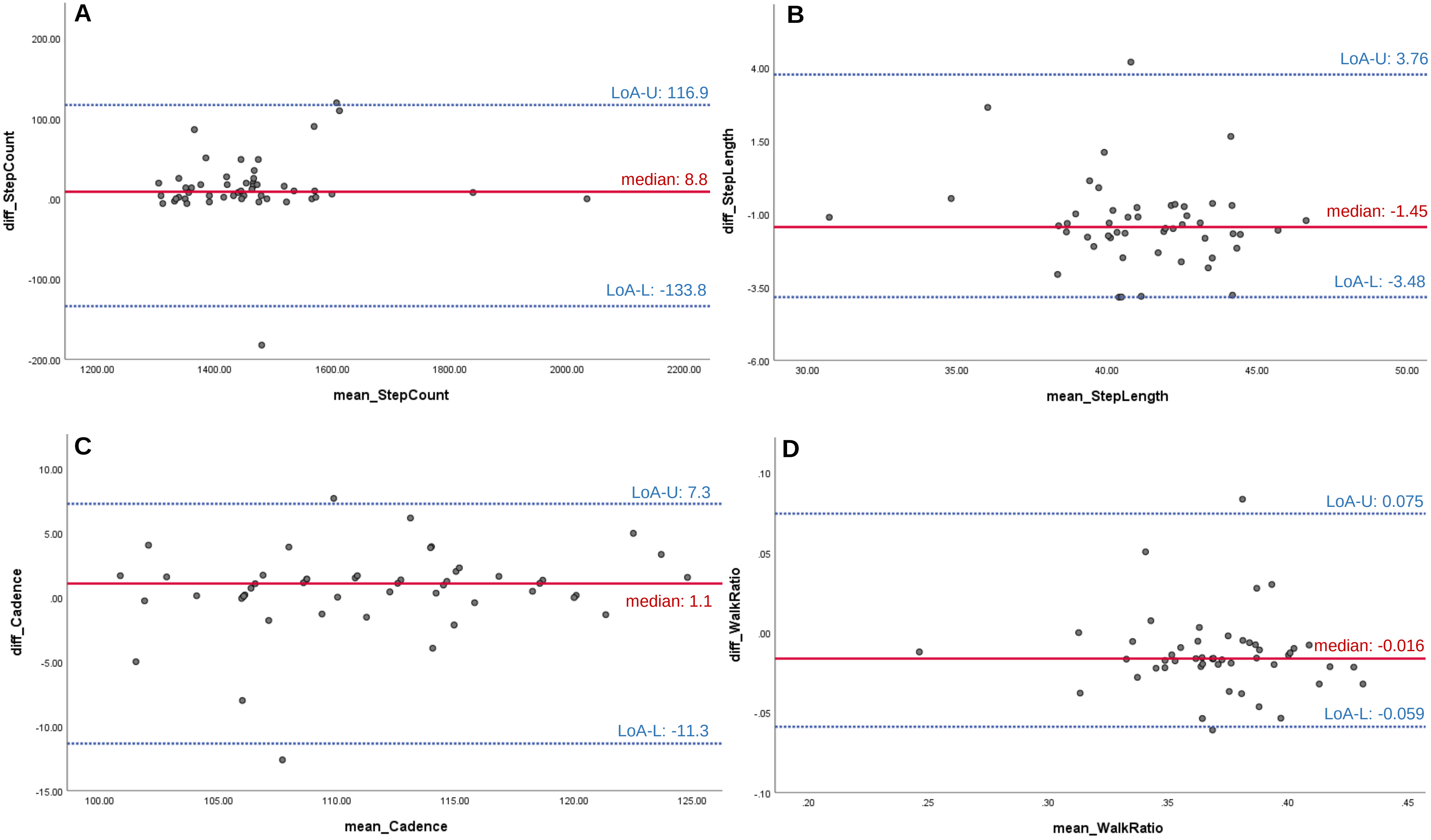

Supplement 3:
Bland-Altman Plots for Agreement with the gold standard in Healthy Participants (N = 50); A: Step Count, B: Step Length, C: Cadence, D: Walk Ratio
diff: difference; LoA-U: upper Limit of Agreement; LoA-L: lower Limit of Agreement

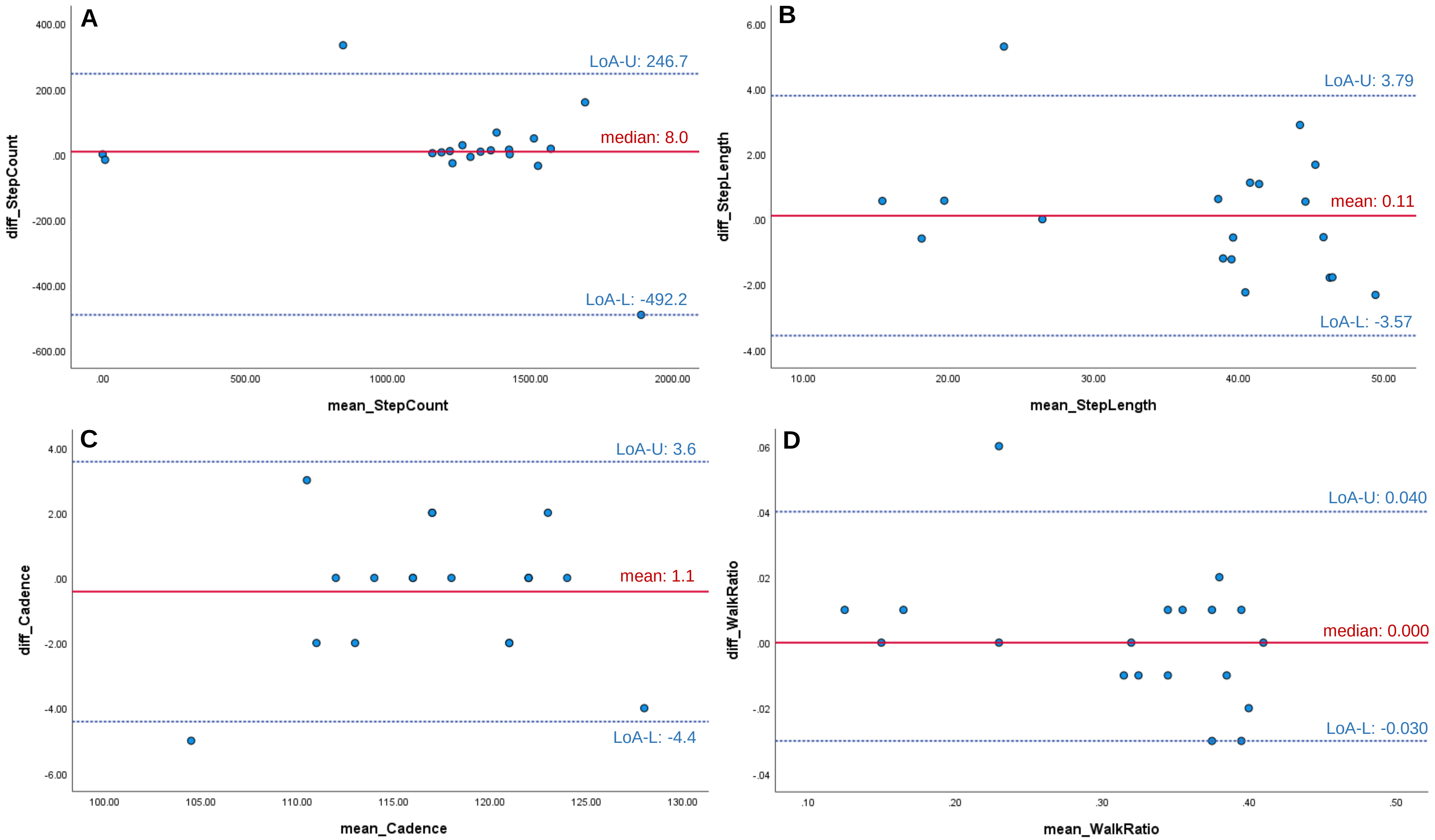

Supplement 4:
Bland-Altman Plots for Test-retest Agreement in Participants with Chronic Stroke (all data, N = 19); A: Step Count, B: Step Length, C: Cadence, D: Walk Ratio
diff: difference; LoA-U: upper Limit of Agreement; LoA-L: lower Limit of Agreement

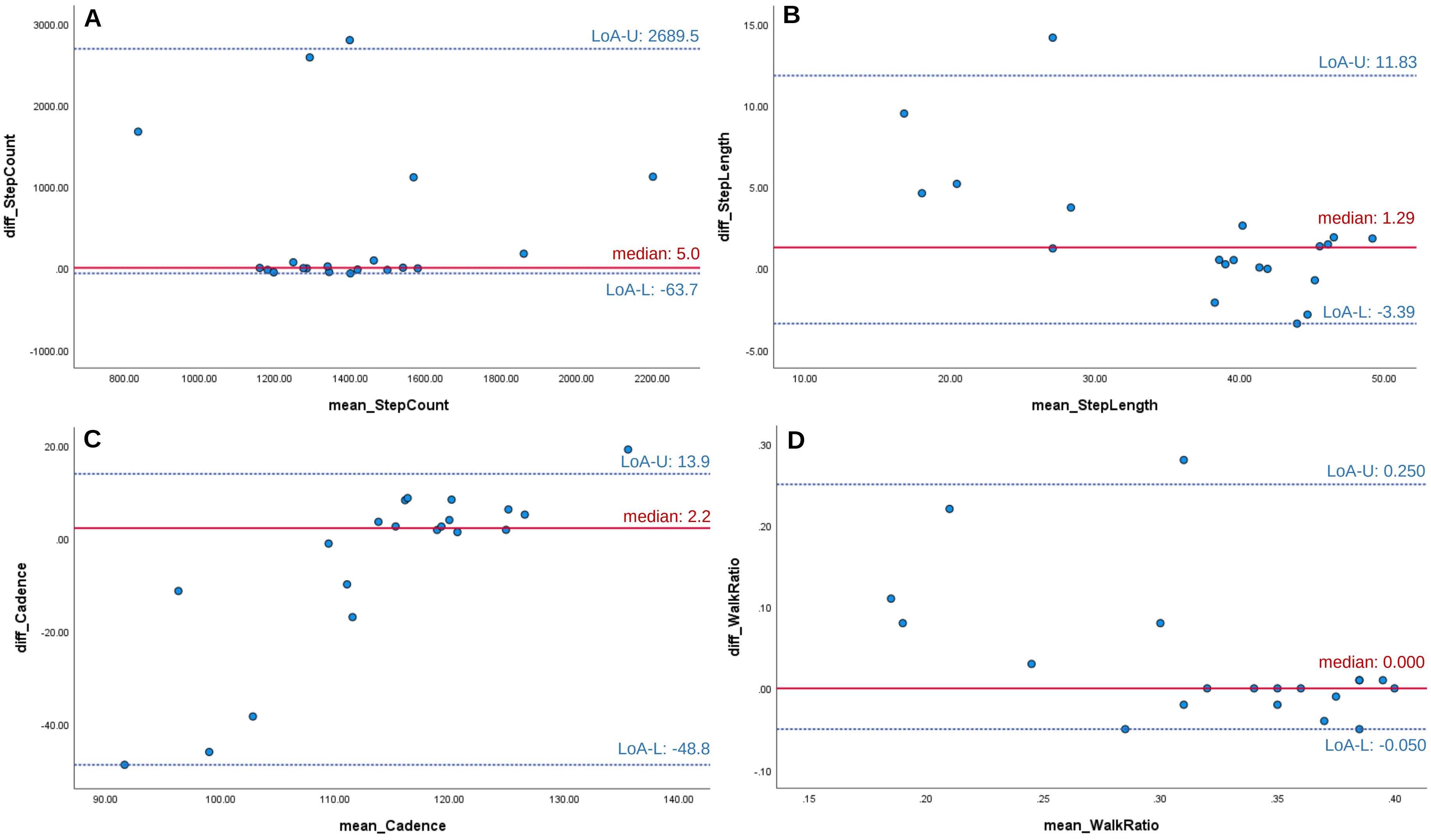

Supplement 5:
Bland-Altman Plots for Agreement with the gold standard in Participants with Chronic Stroke (all data, N = 20); A: Step Count, B: Step Length, C: Cadence, D: Walk Ratio
diff: difference; LoA-U: upper Limit of Agreement; LoA-L: lower Limit of Agreement

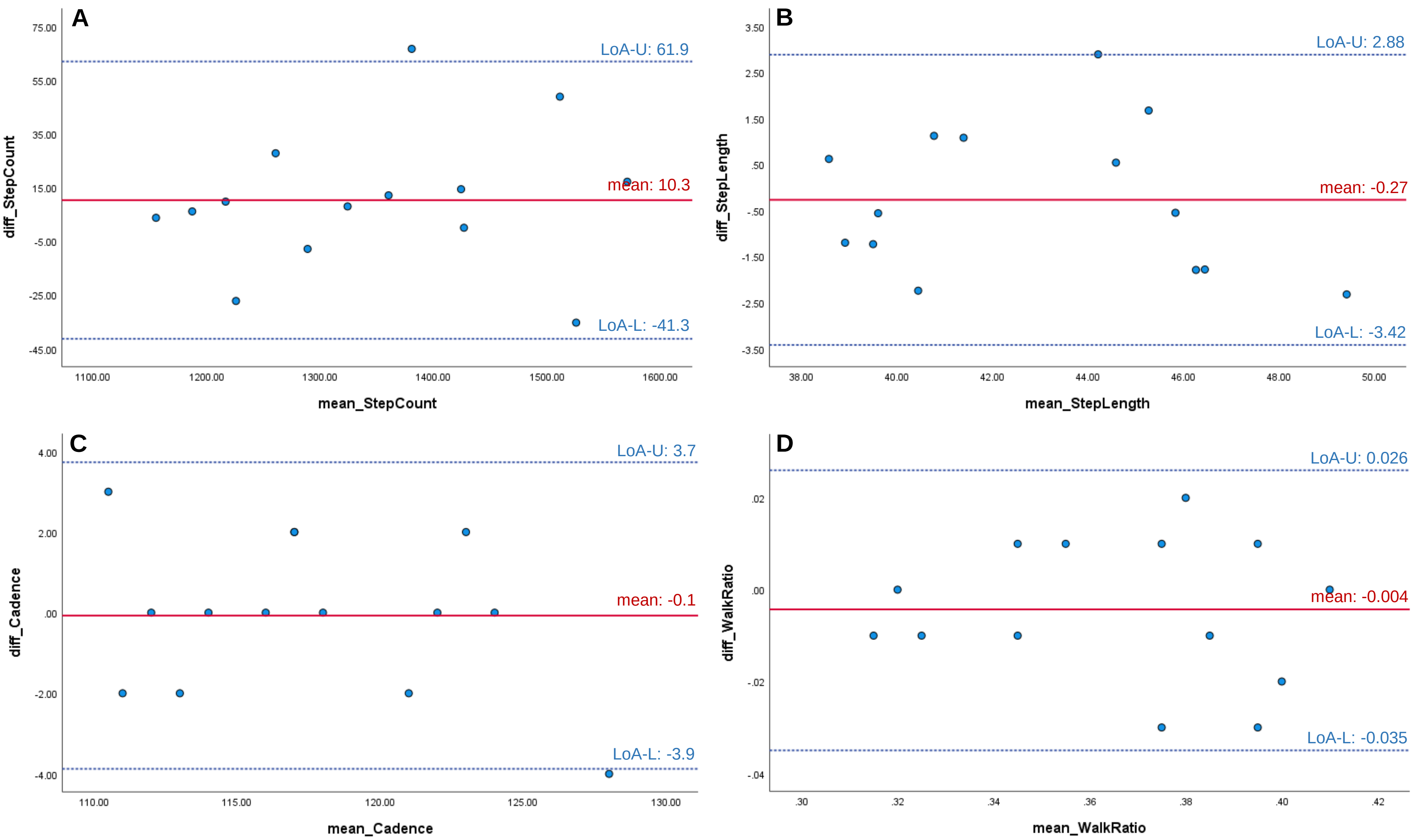

Supplement 6:
Bland-Altman Plots for Test-retest Agreement in Participants with Chronic Stroke (walking < 1m/s excluded, N = 14); A: Step Count, B: Step Length, C: Cadence, D: Walk Ratio
diff: difference; LoA-U: upper Limit of Agreement; LoA-L: lower Limit of Agreement

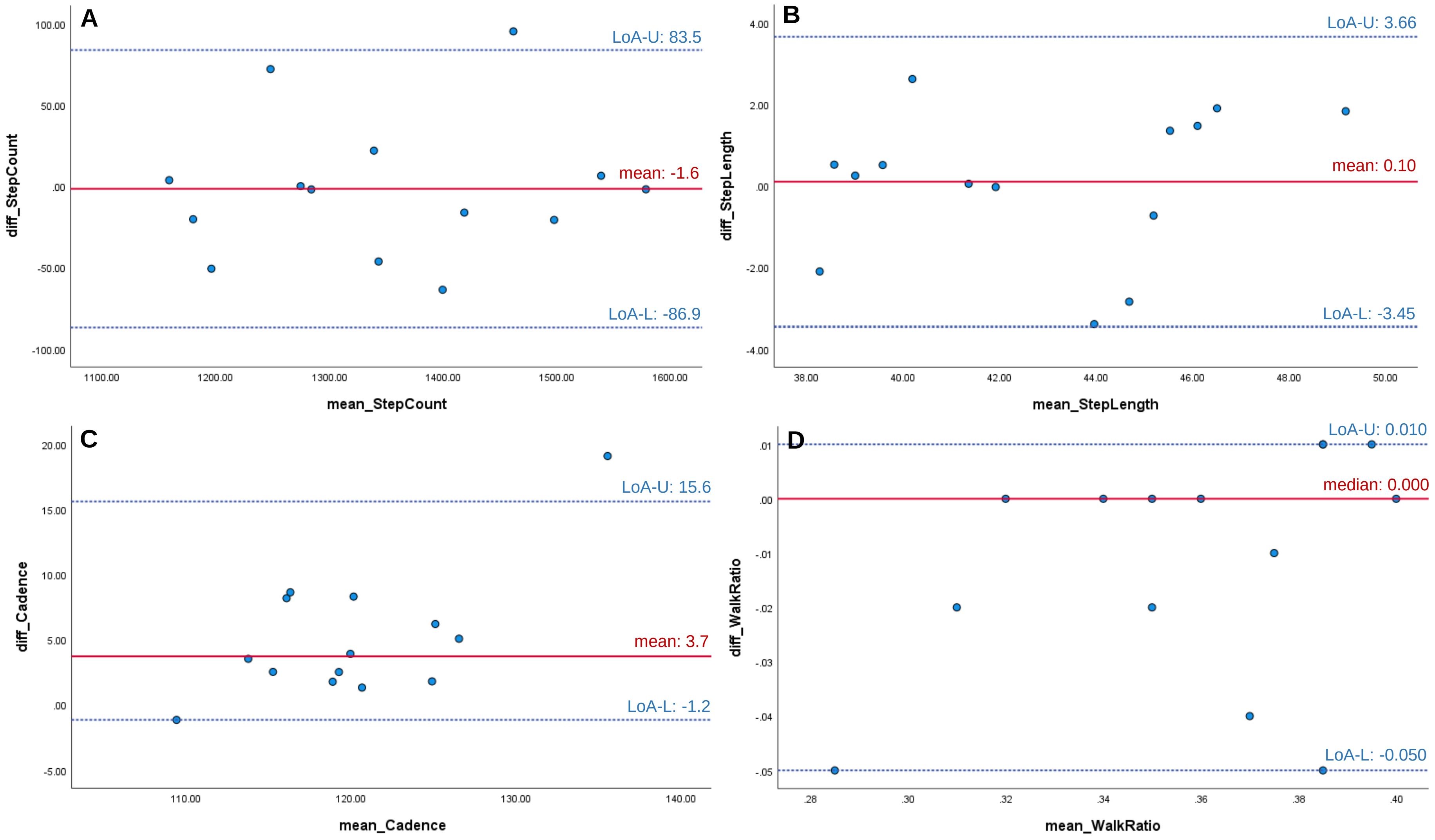

Supplement 7:
Bland-Altman Plots for Agreement with the gold standard in Participants with Chronic Stroke (walking < 1m/s excluded, N = 14); A: Step Count, B: Step Length, C: Cadence, D: Walk Ratio
diff: difference; LoA-U: upper Limit of Agreement; LoA-L: lower Limit of Agreement
