# Supplementary material for: Agreement, Reliability, and Concurrent Validity of an Outdoor, Wearable-Based Walk Ratio Assessment in Healthy Adults and Chronic Stroke Survivors
Source: Front Physiol. 2022 Jun 20;13:857963. doi: 10.3389/fphys.2022.857963 (PMC9252290; doi:10.3389/fphys.2022.857963)
Supplement: Supplementary file 2 [file DataSheet1.PDF]

Health Questionnaire

USZ Universitäts Spital Zürich

ETH Eidgenössische Technische Hochschule Zürich
Swiss Federal Institute of Technology Zurich

Prp. ID: **Date:** **Veryfied:**

*** please leave empty, will be filled out by study staff ***

1. General Questions

Gender: ☐ male ☐ female

Age: ______________________________
Weight: ______________________________
Height: ______________________________

Handedness: ☐ right-handed ☐ left-handed

Number of years of education (School + Professional Education or Studies): _____
Highest Degree of Education:
☐ Primary School ☐ Secondary School ☐ Professional Education
☐ Higher education Institution

Do you drink caffeinated drinks? ☐ yes ☐ no
If yes: I drink on average _____ (amount) units* of caffeinated drinks per day.
*One unit corresponds to a glass or cup (≈ 2dl)

Do you smoke? ☐ yes ☐ no
If yes: I smoke on average _____ (amount) cigarettes per day.

Do drink alcohol? ☐ yes ☐ no
If yes: I drink on average _____ (amount) units* of alcoholic drinks per week.
*One unit corresponds to a glass of wine (≈ 2dl), a small beer, a small amount of high-proof alcohol

2. Questions about your Health

How would you judge your general health at the moment?
☐ excellent ☐ very good ☐ good ☐ not so good ☐ bad ☐ I don't know.

Do you have:
Walking problems: ☐ yes ☐ no
Balance problems: ☐ yes ☐ no
Vertigo: ☐ yes ☐ no

Has a doctor ever diagnosed one of the following diseases in you?

Diabetes mellitus (sugar disease)	☐ yes	☐ no	☐ I don't know
If yes: Disease controlled / Do you take medication?	☐ yes	☐ no	☐ I don't know
Hypertension (High Blood Pressure)	☐ yes	☐ no	☐ I don't know
If yes: Disease controlled / Do you take medication?	☐ yes	☐ no	☐ I don't know
Heart Insufficiency (Heart Weakness)	☐ yes	☐ no	☐ I don't know
If yes: Disease controlled / Do you take medication?	☐ yes	☐ no	☐ I don't know
Polyneuropathy	☐ yes	☐ no	☐ I don't know
Recent Heart Failure	☐ yes	☐ no	☐ I don't know

Stroke	☐ yes	☐ no	☐ I don’t know
Malignant Tumor / Cancer	☐ yes	☐ no	☐ I don’t know
Respiratory Disease	☐ yes	☐ no	☐ I don’t know
Stomach or Intestine Disease	☐ yes	☐ no	☐ I don’t know
Joint Disease (Rheumatism, Arthrosis, Gout)	☐ yes	☐ no	☐ I don’t know
Osteoporosis (Bone Atrophy)	☐ yes	☐ no	☐ I don’t know
Obesity (BMI > 40kg/m2)	☐ yes	☐ no	☐ I don’t know
Dementia	☐ yes	☐ no	☐ I don’t know
Eye Disease	☐ yes	☐ no	☐ I don’t know

If yes, what kind? ____

Do you regularly take medications? ☐ ja ☐ nein

If yes, which?

____

____

____

____

____

____

____

Did you experience pain within the last 4 weeks?

☐ no pain ☐ less often than daily ☐ daily

If you had pain, how severe was it (pain intensity)?

☐ very mild ☐ mild ☐ moderate ☐ severe ☐ very severe

If you had pain, where (localisation)?

☐ back pain
☐ hip pain
☐ joint pain: hip, knee, ankle
☐ muscle pain in the lower body (legs, hips)
☐ others
localisation:

____

3. Questions about your Mobility and Physical Activity

Do you suffer from ailments at your legs (Arthrosis, Knee or Hip Pain, Wounds on the feet, Varicose Veins, or others), that limit your mobility?

☐ yes, currently ☐ yes, always ☐ yes, sometimes ☐ no ☐ I don’t know.

Do you use a walking aid?

☐ none ☐ Cane(s) ☐ Walker ☐ others: ________________

Are you afraid of falling?

☐ no, never ☐ sometime / in certain situations ☐ often ☐ yes, always

If in certain situations, when? ________________________________

How often did you fall in the past six months*:

* A fall means an involuntary landing on the floor (e.g. following a loss of balance)

Amount: ☐ 0 ☐ 1 ☐ >1

Cause (if known): ____

Are you physically active at a <u>vigorous</u> intensity, whereby respiration and heart rate increase strongly (e.g. jogging) for at least 10 minutes?
☐ no ☐ yes, on average on ___ (amount) days a week and
on average approx. __:__ (Hr:Min) per day

Are you physically active at a <u>moderate</u> intensity, whereby respiration and heart rate increase moderately (e.g. brisk walking, gardening, strength training) for at least 10 minutes?
☐ no ☐ yes, on average on ___ (amount) days a week and
on average approx. __:__ (Hr:Min) per day

How much time do you on average spend <u>sitting and lying</u> on a regular day (e.g. sitting at a desk, sitting with friends, Car / Bus / Train riding, Watching TV)?
On average ___:___ (Hr:Min) per day

4. Questions about the Stroke *[only given to participants with stroke]*

Time since stroke:	_____________ months		
Type of stroke:	☐ ischemic	☐ haemorrhagic	☐ other
Affected brain side (if known):	☐ right	☐ left	☐ center / both
Affected brain region or vessel (if known):	________________________________		
Paresis:	☐ yes	☐ no	
- if yes, on which body side?	☐ right	☐ left	☐ both
- still present?	☐ yes	☐ no	
if yes, where?	☐ arm	☐ leg	☐ both
if yes, how severe?	☐ complete	☐ weakened	☐ almost normal
Spasticity:	☐ yes	☐ no	
- if yes, on which body side?	☐ right	☐ left	☐ both
- still present?	☐ yes	☐ no	
if yes, where?	☐ arm	☐ leg	☐ both
if yes, how severe?	☐ severe	☐ moderate	☐ mild
Neglect:	☐ yes	☐ no	
- if yes, on which body side?	☐ right	☐ left	
- still present?	☐ yes	☐ no	
